# A Diagnostic Masquerade: Acute Ileoileal Intussusception in a Premature Neonate

**DOI:** 10.7759/cureus.108977

**Published:** 2026-05-16

**Authors:** Rishika Das, Md Naseem, Ashutosh Sinha, Jaibharat Panwar

**Affiliations:** 1 Pediatrics and Neonatology, Yatharth Super Speciality Hospital, Greater Noida, IND; 2 Pediatric Emergency Medicine, Vardhman Mahavir Medical College (VMMC) and Safdarjung Hospital, New Delhi, IND; 3 Pediatric Surgery, Yatharth Super Speciality Hospital, Greater Noida, IND

**Keywords:** ileoileal intussusception, necrotizing enterocolitis, neonatal intestinal obstruction, neonate, preterm neonate

## Abstract

Neonatal intussusception, though a rare cause of intestinal obstruction in premature infants, is a life-threatening condition with nonspecific manifestations. Its ambiguous presentation often mimics necrotizing enterocolitis, which frequently leads to diagnostic delays that may span several days in this vulnerable group. Timely identification, achieved through strong clinical suspicion and targeted imaging such as ultrasonography, is therefore crucial. Prompt exploratory laparotomy with surgical correction is essential to prevent extensive bowel gangrene, as nonsurgical management options are typically ineffective or unsafe. The high morbidity and mortality associated with delayed intervention highlight the urgent need for heightened awareness and rapid decision-making to optimize outcomes.

## Introduction

Intussusception refers to the telescoping of one segment of the intestine into an adjacent segment, resulting in intestinal obstruction that can rapidly become life-threatening. While it represents the most common cause of intestinal obstruction in infants and children between 6 and 18 months of age, it is exceedingly uncommon in neonates and premature infants, accounting for only 3% of all intestinal obstructions and approximately 0.3% (0%-2.7%) of total intussusception cases [1]. This rarity, compounded by the absence of specific clinical features, often leads to diagnostic confusion with the more common necrotizing enterocolitis (NEC) in newborns [2]. As a result, delays in diagnosis and surgical management are frequent, leading to worse outcomes despite the underlying cause often remaining unclear. We report a case of a late preterm neonate referred to our hospital on day 8 of life with symptoms of intestinal obstruction, later confirmed surgically as primary ileoileal intussusception without an identifiable cause.

## Case presentation

This study discusses an infant born at 35 weeks of gestation and weighing 2,100 g, who was delivered by cesarean section for fetal distress. The birth was complicated by meconium-stained amniotic fluid and perinatal asphyxia. Postnatally, the infant developed severe respiratory distress requiring mechanical ventilation. At eight hours of life, she developed seizures and was started on antiepileptic medication. After two days, she was extubated and placed on noninvasive ventilation.

**Figure 1:**
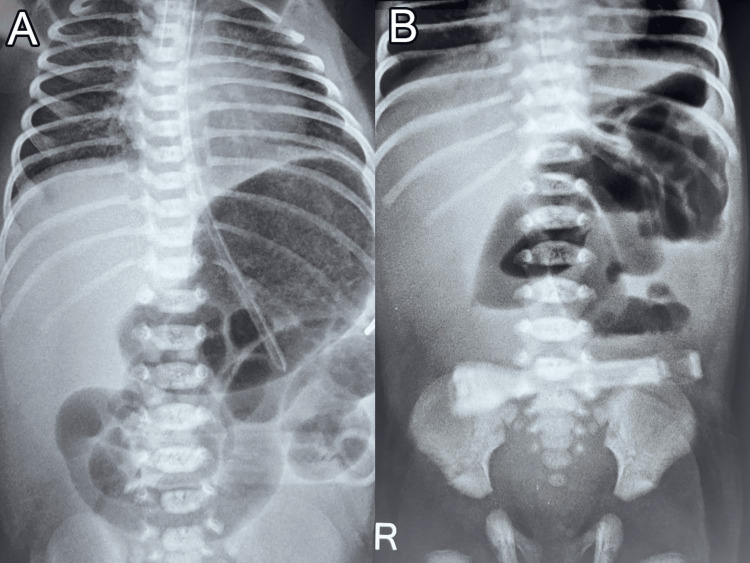
(A) AP view of the abdomen, showing significantly dilated bowel loops. (B) Abdominal X-ray AP view, demonstrating air fluid levels AP: anteroposterior

**Figure 2:**
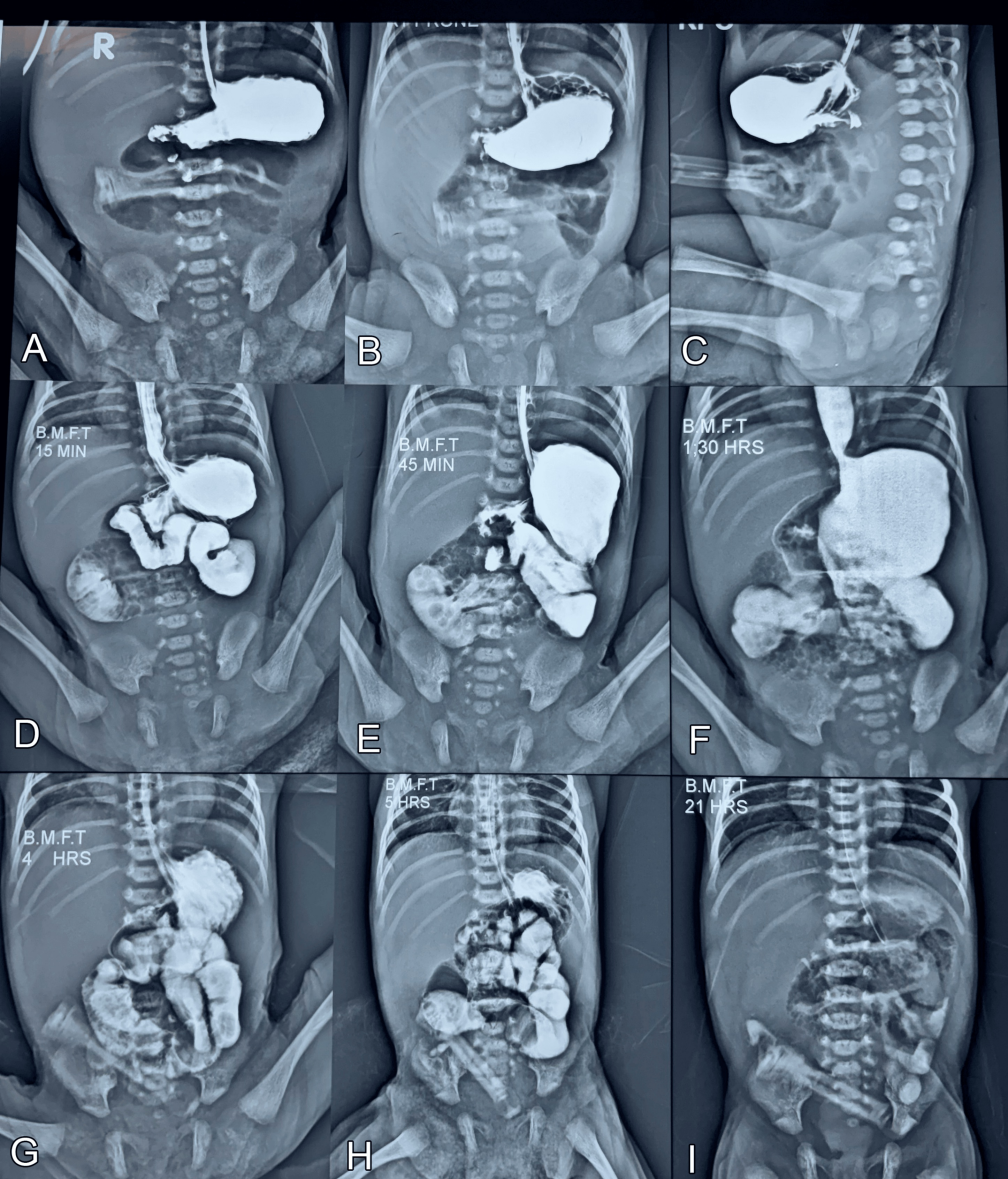
Barium meal follow-through contrast study. This series of radiographic images shows the transit of barium contrast medium through the gastrointestinal tract over a 21-hour period. Supine (A,B) and lateral (C) baseline views prior to the follow-through, showing contrast in the esophagus and stomach. (D) At 15 minutes, contrast has entered the small bowel loops. (E) By 45 minutes, there is progressive filling of the distended proximal small bowel. (F) At 1 hour and 30 minutes, large, dilated small bowel loops in the upper abdomen are filled with contrast. (G) By four hours, there is persistent, severe dilation of the small-bowel loops in the mid-abdomen. (H) At five hours, significant contrast stasis remains in the extensively dilated small bowel. (I) At 21 hours, a small amount of residual contrast is seen in the colon, but marked small bowel dilation is still present, indicative of a severe, chronic-type obstruction

At 48 hours of life, the infant developed bilious vomiting, though small quantities of stool continued to pass. Initial abdominal radiographs showed dilated bowel loops that later progressed to air-fluid levels (Figure 1). A barium meal follow-through conducted at a local facility indicated subacute bowel obstruction (Figure 2). As her condition deteriorated rapidly, accompanied by thrombocytopenia, she was referred to our center for advanced management.

On admission (day 8 of life), the baby had lost 22.7% of her birth weight. Orogastric aspirates were bilious, and the abdomen was soft. She had passed black, blood-stained stool. Laboratory tests revealed an elevated total leukocyte count (19,500/mm³) and a CRP of 98 mg/L, with thrombocytopenia (platelet count 56,000/mm³). Abdominal X-ray showed paucity of bowel gas, suggesting possible bowel necrosis.

The clinical presentation necessitated a broad differential diagnosis focused on neonatal intestinal emergencies, specifically considering mechanical obstructions such as gut malrotation (with or without midgut volvulus), jejunoileal atresias, and meconium ileus, as well as the inflammatory process of NEC. While these conditions can share overlapping symptoms, such as abdominal distension and feeding intolerance, the diagnostic clarity was refined by radiographic evidence. The abnormal persistence of contrast stasis for more than five hours served as a critical indicator of a physical blockage rather than a functional delay. When synthesized with the patient’s evolving clinical status, this prolonged transit time solidified the suspicion of a high-grade mechanical obstruction, ultimately mandating an immediate transition to emergent exploratory laparotomy for definitive surgical intervention.

**Figure 3:**
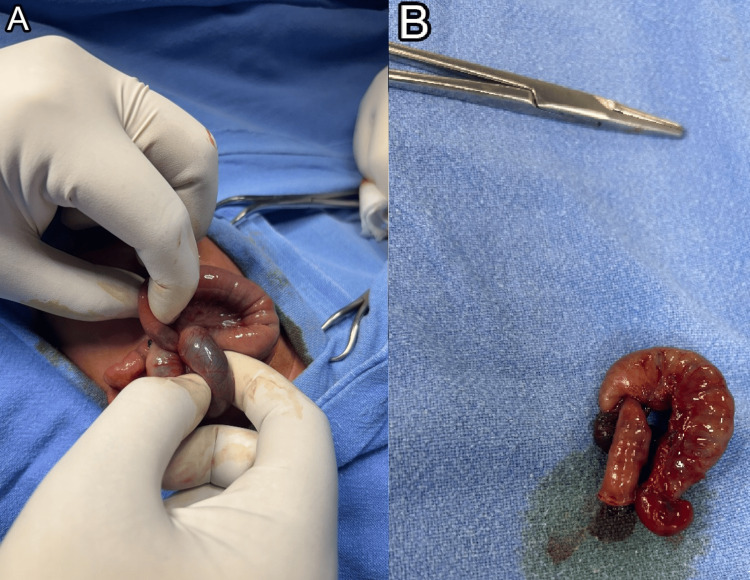
(A) Intraoperative visualization demonstrating an ileoileal intussusception, with a distinct segment of bowel invaginating into the distal lumen. The affected loop exhibits clear signs of vascular compromise and ischemia. (B) Resected specimen showing the isolated segment of the intussusceptum. The bowel wall is severely congested, dusky, and nonviable, confirming segmental gangrene secondary to prolonged strangulation

Owing to the acute clinical findings, the baby was urgently cleared for surgical intervention and underwent an emergent exploratory laparotomy within hours of initial admission. Intraoperatively, a 17-cm segment of gangrenous bowel was identified along with an ileoileal intussusception (Figure 3). The intussusception was reduced, followed by resection of the gangrenous segment and primary ileoileal anastomosis.

Postoperatively, the infant required ventilatory support for one day, followed by two days on a heated, humidified, high-flow nasal cannula before transitioning to room air. Empirical antibiotics were administered for five days. Platelet transfusions (two units of random-donor platelets and one unit of single-donor platelets) led to hematologic improvement. Feeds were resumed on postoperative day 5 and advanced gradually to full feeds by day 8. She was discharged on day 10 after surgery, weighing 2,190 g. She remained stable on follow-up.

## Discussion

Intussusception is a leading cause of intestinal obstruction among infants aged 6-18 months but remains extremely rare in neonates, particularly in premature infants [1].

In preterm neonates, the pattern and pathophysiology of intussusception differ markedly from those in older infants. Whereas ileocolic intussusception accounts for approximately 80% of cases in the typical age group, small bowel involvement is seen in fewer than 10% [1,3,4]. In premature neonates, however, small bowel intussusception predominates, affecting the ileum and jejunum in up to 96.6% of cases, with ileoileal intussusception being the most frequent type [1,3,4]. Consistent with these observations, our case involved ileoileal intussusception.

The etiology of neonatal intussusception remains uncertain, especially in preterm infants [2]. In full-term neonates, a pathological lead point is found in approximately 58% of cases, including anomalies such as duplication cysts, hamartomas, Meckel’s diverticulum, or mesenchymomas [4]. In preterm infants, however, a lead point is identifiable in only about 8% [5]. It is postulated that perinatal factors that cause intestinal hypoperfusion or hypoxia may lead to dysmotility or localized stricture formation, thereby predisposing to intussusception [6]. Inspissated meconium has also been implicated as a mechanical trigger. Overall, hypoxic episodes may play a pivotal etiologic role in late-onset neonatal intussusception.

Symptoms in preterm neonates typically appear around 10 days of age, coinciding with the period when hypoxic-ischemic events from delayed cardiopulmonary adaptation are most common. Diagnosis is notoriously difficult because the clinical picture often mimics NEC. Shared symptoms include abdominal distension, vomiting, feeding intolerance, and bloody stools, while the classical palpable abdominal mass is rarely present. NEC, being more prevalent, is often presumed first, resulting in an average diagnostic delay of 7 days, especially in nonperforated cases [7].

Certain clinical clues, however, may point toward intussusception rather than NEC: persistent bilious aspirates, prominent abdominal distension without pneumatosis intestinalis, and rectal bleeding unaccompanied by systemic toxicity. Radiological findings are often nonspecific. Plain abdominal films typically show dilated loops and air-fluid levels. Contrast enema, while valuable in full-term infants due to frequent ileocolic involvement, is less informative and riskier in preterm neonates given the predominance of small bowel disease and the increased likelihood of perforation [6].

USG remains the most useful imaging modality in this setting. It is radiation-free, capable of identifying lead points, and effective in detecting small bowel intussusceptions [1]. Nevertheless, distended bowel loops or displacement of the sigmoid colon to the right side may obscure the findings, posing a potential diagnostic pitfall.

Prompt recognition and surgical management are essential. While NEC often presents within the first five days of life and quickly leads to systemic deterioration, intussusception usually manifests after the first week, with the infant’s general condition remaining stable until perforation occurs. This distinction makes intussusception an important differential diagnosis when gastrointestinal symptoms persist in an otherwise stable neonate.

Given the poor response to nonsurgical methods, early laparotomy remains the cornerstone of treatment. Primary anastomosis is preferred when feasible, whereas stoma formation may be necessary in unstable or septic patients. Although surgical outcomes have improved, prognosis largely depends on the timing of diagnosis and the infant’s underlying comorbidities. As this case illustrates, a delay in intervention markedly worsens survival outcomes.

## Conclusions

Intussusception, though rare in neonates, represents a critical diagnostic challenge due to its clinical similarity to NEC and volvulus. Misinterpretation or delayed recognition can significantly increase morbidity and mortality. Careful attention to subtle clinical and radiologic distinctions is vital to prevent delays in treatment. Maintaining a high index of suspicion and acting swiftly can be lifesaving in this rare but serious condition.
